# A Review on Rainfall Measurement Based on Commercial Microwave Links in Wireless Cellular Networks

**DOI:** 10.3390/s22124395

**Published:** 2022-06-10

**Authors:** Bin Lian, Zhongcheng Wei, Xiang Sun, Zhihua Li, Jijun Zhao

**Affiliations:** 1 School of Water Conservancy and Hydroelectric Power, Hebei University of Engineering, Handan 056038, China; lianbin@hebeu.edu.cn; 2 Hebei Key Laboratory of Intelligent Water Conservancy, Hebei University of Engineering, Handan 056038, China; 3 School of Information and Electrical Engineering, Hebei University of Engineering, Handan 056038, China; weizhongcheng@hebeu.edu.cn (Z.W.); lizhihua@hebeu.edu.cn (Z.L.); 4 Hebei Key Laboratory of Security & Protection Information Sensing and Processing, Hebei University of Engineering, Handan 056038, China; 5 Department of Electrical and Computing Engineering, University of New Mexico, Albuquerque, NM 87131, USA; sunxiang@unm.edu

**Keywords:** wireless cellular networks, microwave links, rainfall measurement, machine learning, remote sensing

## Abstract

As one of the most critical elements in the hydrological cycle, real-time and accurate rainfall measurement is of great significance to flood and drought disaster risk assessment and early warning. Using commercial microwave links (CMLs) to conduct rainfall measure is a promising solution due to the advantages of high spatial resolution, low implementation cost, near-surface measurement, and so on. However, because of the temporal and spatial dynamics of rainfall and the atmospheric influence, it is necessary to go through complicated signal processing steps from signal attenuation analysis of a CML to rainfall map. This article first introduces the basic principle and the revolution of CML-based rainfall measurement. Then, the article illustrates different steps of signal process in CML-based rainfall measurement, reviewing the state of the art solutions in each step. In addition, uncertainties and errors involved in each step of signal process as well as their impacts on the accuracy of rainfall measurement are analyzed. Moreover, the article also discusses how machine learning technologies facilitate CML-based rainfall measurement. Additionally, the applications of CML in monitoring phenomena other than rain and the hydrological simulation are summarized. Finally, the challenges and future directions are discussed.

## 1. Introduction

Rainfall is one of the major driving forces in the hydrological cycle of the land area. Natural disasters caused by rainfall, such as flash flood, debris flows, landslides, and urban waterlogging, have been damaging people’s lives and property over the years [1]. On 20 July 2021, floods and secondary disasters caused by torrential rain had a negative impact on 14.786 million people in Zhengzhou, China, with a direct economic loss of 120.06 billion yuan [2]. Therefore, developing rapid and accurate rainfall information acquisition technologies is extremely urgent and important to achieve precise flood risk assessment and enhance the public safety response capability. Meanwhile, rainfall is one of the most critical climate factors to determine crop growth, and accurate precipitation information can substantially improve crop yield [3]. In addition, rainfall information is also an important reference index for reservoir operations, urban water supply, irrigation planning, and other forms of water resource management [4]. However, due to the high spatial and temporal dynamics of rainfall, it is still challenging to accurately monitor and measure rainfall in real time [5,6].

Rain gauges (RGs), weather radars, and satellite remote sensing are the three main rainfall measurement methods at present. RG is a point measurement tool with limited density, low spatial resolution, and relatively high construction and maintenance costs. Radar observation of rainfall emits electromagnetic waves into the sky. These electromagnetic waves are reflected by clouds and returned to a ground receiver. Rainfall information is then estimated by comparing the original and returned electromagnetic waves [7]. This method measures the water volume in the clouds, which could be quite different from the rainfall amount on the ground. In addition, this method can be easily affected by ground obstacles at a low elevation angle [8]. Satellite rainfall measurement, on the other hand, has a global scale, but the resolution is relatively coarse in terms of small spatial and temporal scales. Moreover, its accuracy is significantly affected by the density of clouds, and the time lag is high [9].

In the recent years, many countries such as Israel, the Netherlands, France, Germany, the Czech Republic, Switzerland, Italy, and China have adopted commercial microwave links (CMLs) in wireless cellular networks (WCNs) to retrieve the regional rainfall information. The measurement accuracy and ability of capturing the spatial and temporal dynamics of rainfall for the CML method have been verified through a variety of simulations and experiments. The CMLs that have already been deployed in WCNs to achieve fronthaul/backhaul communications among base stations can be considered as a widely distributed environmental monitoring sensor network. Since CMLs were originally designed for the communications purpose and environment sensing is just a by-product, the network of CMLs is also called an opportunistic wireless sensor network (OWSN) [10]. The IMT-2030 (6G) Promotion Group released a research report on the integration of communications and sensing technology in September 2021 [11], which points out that the communications and sensing capabilities will finally achieve the integration and symbiosis in the future 6G wireless networks. According to the statistics of the Industry and Information Technology Ministry, the number of 5G base stations in China has already exceeded one million in 2021. The increasing number of the deployed base stations and microwave links provides great opportunities and supports to develop CML-based rainfall measurement technology [12,13,14]. Table 1 provides the comparisons of the existing rainfall measurement methods.

In WCNs, in order to monitor the quality of service (QoS) of a CML, the network system always regularly monitors and records the transmission and received power of the signal in the CML for every, for example, 15 min. That is, a huge amount of CML attenuation data is already available and can be used to retrieve real-time rainfall intensity. However, there are many factors leading to the signal attenuation during the propagation, and it is nontrivial to extract the rain-induced attenuation from these attenuation data. This is because, first, microwave link attenuation data contain many spatio-temporal variations and uncertainties, which are not only related to path length, frequency, polarization, and other attributes of microwave links [15], but also affected by external factors such as temperature, humidity, atmospheric composition, and so on; second, rainfall is a highly spatio-temporal dependent event [16], and it is relatively complex and difficult to establish the model between rain-induced attenuation and rainfall intensity. Considering the problems mentioned above, data-driven methods such as machine learning have been proposed to facilitate CML-based rainfall measurement. Machine learning is a new way to solve interdisciplinary issues and the problems which are difficult to simulate their processes [17]. In particular, deep learning (DL) has been proven to be an important tool to exploit the power of big data (BD) by capturing high-dimensional and multi-modal data distribution, and extracting spatio-temporal features of data in order to understand the internal data logic and whole physical process [18].

Rainfall measured by the CML network has a broad application prospect in both densely populated cities and remote mountainous areas. Although current studies have made significant contributions on the technological aspects, there are very few review articles about this technique. One study [1] reviews the existing technologies and challenges of CML-based precipitation monitoring from a signal processing prospective, which includes calibration, detection, estimation, classification and separation, assimilation, and reconstruction. Another [19] divides the CML-based measurement studies into four groups and points out that the biggest challenge is the capitalization of CML data. Overviews of the history, theory, challenges, and opportunities toward CML-based rainfall monitoring technique are given by refs. [5,10]. The former focuses on the background introduction and continental-scale rainfall monitoring, while the latter emphasizes the issues of wet antenna attenuation and abnormal signal fluctuations. Studies related to terrestrial microwave rain attenuation measurement from 2010 to 2020 are investigated by [20], but the detailed analyses of different methods and current challenges are insufficient. All of the above literature reviews summarize current research either from a certain prospective or focus on specific problems, but a comprehensive literature review and comparisons have not been carried out. Thus, it is paramount important to give a thorough review on this promising rainfall measurement technology; in particular, to summarize the literature of the past 2 years, in which machine learning has been developing rapidly and has been applied to this realm widely. The main contributions of this paper are as follows:

- The paper illustrates the main steps in CML-based rainfall measurement, summarizing the state-of-the-art solutions in each step.
- The paper analyzes uncertainties and errors involved in CML-based rainfall measurement as well as their impacts on the measurement accuracy.
- The paper explores the existing machine learning methods to facilitate CML-based rainfall measurement. To the best of our knowledge, this paper provides the first comprehensive review on machine learning for CML-based rainfall measurement.
- The paper summarizes the open-access datasets and codes related to CML-based rainfall measurement, and discusses the current challenges and future directions.

The rest of the paper is organized as follows. Section 2 describes the principle and theory of CML-based rainfall measurement. Section 3 outlines the revolution of CML-based rainfall measurement. The steps from CML received signal level (RSL) to rainfall map are presented in Section 4. The machine learning solutions to facilitate measurement are discussed in Section 5. Monitoring phenomena other than rain and the hydrological applications are explored in Section 6 and Section 7, respectively. The existing challenges and future directions are presented in Section 8, and finally, Section 9 draws a conclusion.

## 2. The Principle and Theory of Rainfall Measurement by CML

### 2.1. Basic Principle

**Figure 1:**
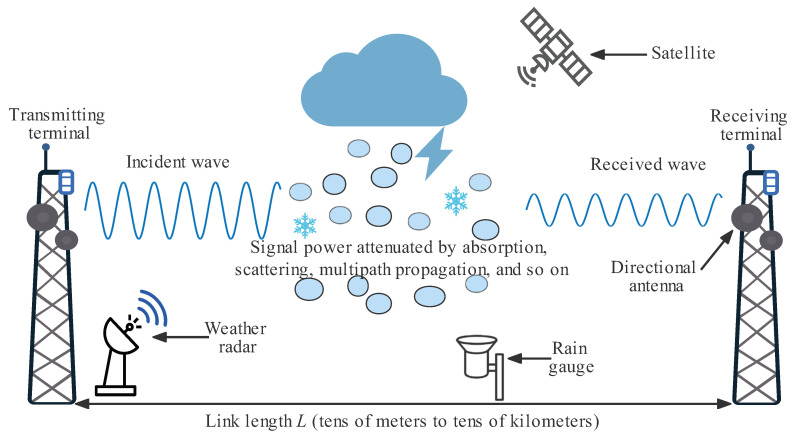
Basic operating principle of CML-based rainfall measurement.

Wireless communications use electromagnetic waves, which can propagate in free space, to carry data. Microwave refers to the electromagnetic wave with 300 MHz–300 GHz frequency, i.e., 1 mm–1 m wavelength. Microwave can be further divided into decimeter wave, centimeter wave, and millimeter wave. Normally, round directional antennas have stronger beam focusing characteristics than rectangular omnidirectional antennas. The communication link between two such directional antennas is called a microwave backhaul link, or microwave link for short. As shown in Figure 1, when the microwave propagates through the rain area, the transmitting power will be attenuated due to the scattering and absorption by raindrops [21,22]. This type of microwave attenuation is called rain-induced attenuation, and the relative loss of power per unit path length is called specific attenuation, denoted as *k* (dB/km). Recommendation ITU-R P.838-3 [23] gives the relationship between the specific attenuation *k* and rain rate, denoted as *R* (mm/h), as follows:

(1) $$k=aR^{b},$$

where the coefficients *a* and *b* are functions of microwave signal frequency, polarization, and raindrop size distribution (DSD). In addition to rain-induced attenuation, non-rain factors, such as free space path loss, atmospheric attenuation and multipath effect, can also lead to microwave link attenuation. Therefore, the rain-induced attenuation can only be obtained by eliminating the non-rainfall induced attenuation from the total attenuation. CML-based rainfall measurement has the advantages of path-integrated measurement, high spatial density, less human intervention, low implementation cost, and true reflection of near-surface rainfall and thus can be used as an effective substitution or supplement to traditional rainfall measurement methods, such as RGs, weather radar, and satellite [20,24,25]. In addition, CML-based measurement can estimate not only rainfall, but also water vapor, solid particles, fog, snow, sleet, hail, and so on [26,27,28,29].

### 2.2. Mathematical Models

The amount of signal attenuation, denoted as $A_{tot}$ (dB), is obtained by subtracting the received power from the transmitting power. Here, $A_{tot}$ comprises three types of attenuations, i.e., baseline attenuation $A_{baseline}$ (dB), path-integrated rain attenuation $A_{rain}$ (dB), and wet antenna attenuation $A_{WAA}$ (dB). That is,

(2) $$A_{tot}=A_{baseline}+A_{rain}+A_{WAA}.$$

#### 2.2.1. Baseline Attenuation

$A_{baseline}$ is usually determined by the attenuation value in a time interval right before a rainfall event and mainly consists of free space path loss $A_{bf}$ (dB) and atmospheric attenuation $A_{gas}$ (dB), i.e.,

(3) $$A_{baseline}=A_{bf}+A_{gas}.$$

- Free-space path loss $A_{bf}$. The free-space path loss is the loss of signal strength when a signal propagates through free space. Free-space path loss $A_{bf}$ increases as the distance between the transmitter and the receiver increases. According to Recommendation ITU-R P.525-4 [30], when the distance between antennas is much larger than the electromagnetic wavelength λ, the path loss of electromagnetic wave in free space is only related to the frequency and distance, as shown in Equation (4):
  (4) $$A_{bf}\;=20\;log\left(4\pi L/\lambda \right)=32.4+20\;logf+20\;logL,$$
  where *f* (MHz) is carrier frequency and *L* (km) is the distance between the transmitter and the receiver.
- Atmospheric attenuation $A_{gas}$. When electromagnetic waves propagate through the atmosphere, they will be attenuated by the absorption, reflection, and scattering of water vapor, fog, solid particles, oxygen, nitrogen, carbon dioxide, and other substances in the atmosphere. According to Recommendation ITU-R P.676-12 [31], the specific gaseous attenuation of a microwave link, denoted as $k_{gas}$ (dB/km), can be estimated by
  (5) $$k_{gas}=k_{o}+k_{w}=0.1820f\left(N_{oxygen}^{\prime \prime }\left(f\right)+N_{watervapor}^{\prime \prime }\left(f\right)\right),$$
  where $k_{o}$ and $k_{w}$ (dB/km) are the specific attenuation caused by dry air (oxygen, nitrogen, etc.) and water vapor, respectively, and $N_{oxygen}^{\prime \prime }$ and $N_{watervapour}^{\prime \prime }$ are the imaginary parts of the frequency-dependent complex refractivities. Then, the atmospheric attenuation of a microwave link can be estimated by
  (6) $$A_{gas}=\int_{0}^{L}k_{gas}\left(l\right)dl.$$

#### 2.2.2. Path-Integrated Rain Attenuation

According to refs. [5,10,19], path-integrated rain attenuation $A_{rain}$ in Equation (2) basically is a function of the specific attenuation *k*, which can be calculated based on Equation (1), that is,

(7) $$A_{rain}=\int_{0}^{L}k\left(l\right)dl=\int_{0}^{L}aR{\left(l\right)}^{b}dl\overset{b\approx 1}{=}a{\bar{R}}^{b}L,$$

where *R* (mm/h) is the path-average rain rate. The attenuation caused by rainfall and the average rain rate are linearly correlated if *b*≈ 1. Other than Equation (1), the values of the rain rate *R* and the specific attenuation *k* can be estimated based on DSD refer to [32,33], i.e.,

(8) $$R=6\times 10^{-4}\pi \int_{D}v\left(D\right)D^{3}N\left(D\right)dD,$$

(9) $$k=\frac{1}{ln(10)}\int_{D}Cext(D,f)N(D)dD,$$

where *D* is the raindrop diameter in mm, N(D) is the DSD’s number concentration per diameter in mm^−1^m^−3^, v(D) is the rain droplet final velocity in m/s, and Cext(D,f) is the extinction cross section at frequency *f* in m^2^, which describes the attenuation of the signal at frequency *f* by each raindrop. The dependence of Cext(D,f) and $v\left(D\right)D^{1}$ on raindrop diameter *D* is very similar, especially in the frequency range from 20 GHz to 35 GHz [5].

#### 2.2.3. Wet Antenna Attenuation (WAA)

WAA ($A_{WAA}$) refers to the signal attenuation due to the water film attached to the radome during the rainfall, and it will last for a period of time until the water on the radome evaporates. Because $A_{baseline}$ is determined prior to the rainfall event, WAA is generally not included in $A_{baseline}$. Usually, WAA is assumed as a constant value (1–2 dB) or determined by the RSL time series of the link and its surrounding links in the previous time, and the time probability distribution function of WAA can also be estimated by data-driven algorithms.

### 2.3. Example Demonstration

**Figure 2:**
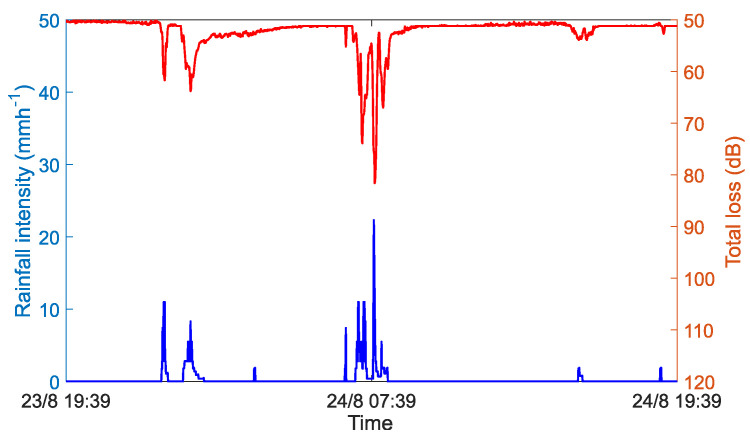
Correlation between CML power total loss and rainfall intensity.

Figure 2 shows the relationship between the total attenuation of the CML and the rainfall intensity measured by RG. The simulation data come from an open access dataset [34], which contains the signal power total loss collected from six E-band (71–76 GHz and 81–86 GHz) CMLs and the rainfall intensity collected from four RGs in Prague, Czech Republic. We selected the data from one CML of the six and one RG, which is nearest to the selected link midpoint, to generate the signal level time series of 24 h (23 August 2018 19:39–24 August 2018 19:39) with a time resolution of 1 min. It can be seen that there is a clear correlation between CML power total loss and rainfall intensity, and even very low intensity rainfall can be perceived by the CML measurement, because the E-band CML is more sensitive to rainfall than other used frequency bands (roughly from 10 to 50 GHz) at present [35,36].

## 3. Development of Rainfall Measurement Based on CML

In the middle of the 20th century, it was found that rainfall had a significant impact on the attenuation of electromagnetic waves, especially high-frequency electromagnetic waves, and it was proposed that the attenuation of electromagnetic waves could be used to retrieve rainfall intensity [37]. At the end of the 20th century, the implementation of the Tropical Rainfall Measuring Mission (TRMM) project has promoted a series of experiments to retrieve DSD by using dedicated dual-frequency links [38]. However, it is not realistic to deploy such a network of microwave links just for rain measurement in a large area. Fortunately, since the beginning of the 21st century, with the rapid growth number of mobile phone users, a variety of wireless base stations and microwave links have been widely deployed to build seamless WCNs. This forms the infrastructure foundation of the CML-based rainfall monitoring networks to timely observe rainfall with high temporal and spatial resolution. Each microwave link between two base stations is equivalent to a sensor node. Moreover, for the typical frequencies used by CML in WCNs, the coefficient *b* in Equation (1) is very close to 1, which means that there is a good linear relationship between rain-induced attenuation and rainfall intensity. In 2006, Messer et al. [39] used CML-RSL data to retrieve the rainfall intensity in Israel for the first time. In 2013, Overeem et al. [40] used an unprecedented number of more than 2400 CMLs to reconstruct the rainfall map for the entirety of the Netherlands (35,500 km^2^). Subsequently, countries around the world established research teams and tried to explore the potential of CML in the field of environmental monitoring. Table 2 summarizes the countries that have achieved rainfall measurement by CML in chronological order.

## 4. Signal Processing from RSL to Rainfall Map

**Figure 3:**
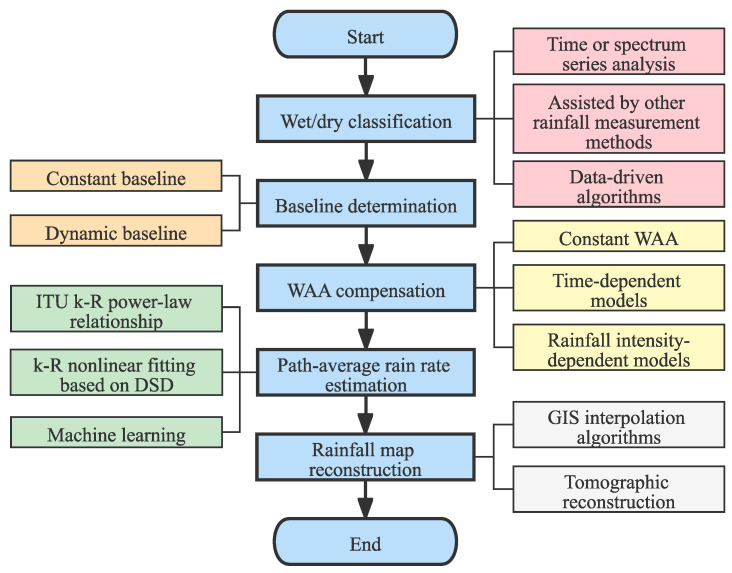
Typical steps in CML-based rainfall measurement.

The main steps from obtaining the RSL data to the generation of rainfall map include the identification of the starting and ending time of rainfall (wet/dry classification), the baseline determination, the compensation of WAA, the calculation of path-average rain rate, and the reconstruction of rainfall map. As shown in Figure 3, each step can be implemented in different ways. The function of each step and the related methods will be described in detail below, and the sources of uncertainty and the reasons to cause errors are also analyzed.

### 4.1. Wet/Dry Classification

After obtaining the RSL data, the first step is to identify the corresponding duration of rainfall from the original rough RSL data. As long as the time period of rainfall is identified, the attenuation for a microwave link during the corresponding time period can be more accurately evaluated to calculate the rainfall intensity. At present, various wet/dry classification methods are widely used to identify the rainfall period and can be mainly categorized into three types.

#### 4.1.1. Time or Spectrum Series Analysis

Based on the assumption that the correlation of RSL time series between two frequencies is higher in the rainy period than in the dry period, Overeem et al. [53] proposed the nearby link approach (NLA). If the RSL value of a microwave link decreases in a time interval and the RSL values of at least half of all the neighbouring links within a radius of 15 km also decrease, then the selected time interval is considered to be wet. Schleiss et al. [42] applied the local variability of the link signal to distinguish the wet and dry weather. Basically, the standard deviation of the RSL data, which are collected within a predetermined 15–30 min window, is calculated, and so the weather during this time period would be regarded as wet if the standard deviation is greater than a predefined threshold. Based on the assumption that rainfall would cause more generation of high-frequency RSL samples, Chwala et al. [43] analyzed the power spectrum of RSL time series based on STFT to determine the wet/dry weather. Wang et al. [54] used the Markov model to distinguish wet/dry period.

#### 4.1.2. Assisted by Other Rainfall Measurement Methods

Rainfall periods can be identified with the help of using rainfall data measured by RGs, radar, or satellite [55]. For example, path-averaged mean 15 min rainfall intensity along the link from unadjusted radar data can be used to identify wet and dry weather conditions for each link and time step. If the rainfall intensity is greater than 0.1 mm/h, the current and the following steps are classified as wet [56].

#### 4.1.3. Machine Learning Algorithms

Song et al. [57] used support vector machine (SVM) to distinguish the wet/dry period. Specifically, the average, minimum, and maximum attenuation are used as the feature vectors, and the radial basis function is used to convert the data. It is pointed out that the classification accuracy increases as the carrier frequency and the length of the link increases. Other machine learning algorithms, such as convolutional neural networks (CNNs) and LSTM, can also be used to establish classification models based on the RSL data, and we will discuss these algorithms in Section 5, which covers machine learning.

In addition, the wet/dry classification methods in current studies also include Fisher discriminant analysis based on kernel function [58] and multi-family likelihood ratio test [59].

### 4.2. Baseline Determination

Baseline attenuation, also known as zero-level attenuation, is the attenuation of the total attenuation excluding the rain-induced part. Baseline attenuation is usually equal to the attenuation of the last dry interval before rainfall occurs. The NLA method takes the median signal level of all the dry periods over the past 24 h as the baseline. Fenicia et al. [60] compared the constant baseline model and the single parameter model based on the first-order low-pass filter, and the results show that the latter model has better performance than the former, but the uncertainty of the latter model is relatively larger during the light rain. Ostrometzky et al. [61] used the minimum attenuation value of CML-RSL to determine the dynamic baseline, which assumes that the rain rate is a random process, the non-rain-induced attenuation is relatively stable in a specific time period, and the real-time dynamic baseline is derived by analyzing the statistical characteristics of the minimum attenuation samples and the rain rate without the need of wet/dry classification in advance.

### 4.3. WAA Compensation

When rainfall occurs, the radome will be covered with a layer of water film to absorb and scatter the signal, which is called wet antenna attenuation [62]. After the rain stops, the water droplets covering the antenna slowly evaporate over time, and so the WAA value gradually decreases until water droplets disappear. If WAA is not taken into account in the CML-based rainfall retrieval, the rain rate will be overestimated. WAA is related to radome material characteristics, temperature, and rain intensity, and WAA in the short link scenario is more significant. Fencl et al. [63] used eight short CMLs at 38 GHz frequency band to quantify WAA, and the experimental results confirm that WAA is closely related to rainfall intensity, and the WAA value is 1.5–2.0 dB in light rain (R < 2 mmh^−1^), 2.8–5.3 dB in rainstorm, and 6–9 dB during heavy rainstorm (R ≈ 70–130 mmh^−1^). Pastorek et al. [64] investigated the performance of six WAA models and the transportability of WAA model parameters among CMLs, which have different features. The results show that the WAA model derived from the rainfall intensity is better than constant WAA model and the time-dependent WAA model. Moreover, the rainfall-intensity-based WAA model does not depend on the frequency and link length, and so it can be reused by other CMLs, where the antenna characteristics are similar.

### 4.4. Path-Average Rain Rate Estimation

After wet/dry classification, baseline determination, and WAA compensation, the rain-induced attenuation in the rainy period is derived, and then the path-average rain rate can be calculated according to Equation (7). The longer the CML link, the greater the attenuation caused by rain on the path, which alleviates the difficulty of low rain rate detection. In addition to the ITU *k-R* model in Equation (1), rain rate can also be estimated by DSD or data-driven algorithms. Song et al. [32] calculated the specific attenuation and rainfall intensity based on DSD, and then the relationship between specific attenuation and rainfall intensity was obtained by using nonlinear fitting. Han et al. [33] also confirmed that the coefficients *a* and *b* in *k-R* relationship of stratiform and convective rain can be estimated based on local DSD measurement, and compared to the coefficients in the ITU-R P.838 document, the derived coefficients achieved an improved rain rate estimation. DL has outstanding advantages in solving the problem of complex dependent data association mining. It can learn the relationship between the input parameters of multi-attribute features and the real-time output rainfall intensity, so as to directly establish the relationship model between CML total attenuation and rainfall intensity. By taking the advance of DL, Habi et al. [65] designed a network structure based on gated recurrent unit (GRU) in recurrent neural network (RNN) to evaluate the rainfall retrieved by CMLs. As compared to the traditional ITU model in Equation (1), the GRU-RNN based model has better performance in terms of lower RMSE and bias, but higher complexity and poorer robustness. A trade-off between robustness and performance can be optimized by introducing a time normalization (TN) layer into the GRU-RNN model. The ability of data-driven methods based on GRU to relate attenuation and rain rate, which can overcome the uncertainties in short links, has been proven by [66].

### 4.5. Rainfall Map Reconstruction

Interpolation algorithm and tomographic reconstruction technology are mainly used to generate rainfall map from path-average rain rate [67,68]. Inverse distance weighting (IDW) and ordinary kriging (OK) are two of the most common spatial interpolation algorithms for a geography information system (GIS) [69,70]. IDW is a fairly simple and robust spatial interpolation point rainfall measurement method. In IDW, if a rainfall intensity value for a given location needs to be predicted, the points closer to the predicted location have a greater impact on it, and so the weight of the surrounding points is set based on the distance to the predicted location, i.e., a short distance means higher weight for a surrounding. The OK algorithm requires enough statistical information of the sample field. Kriging is very suitable for interpolating highly irregular data points. However, OK has its own limitations and needs to make some assumptions, such as isotropy and statistical stationarity. Eshel et al. [71] compared the performance of the IDW and OK algorithms, and the results show that the performance improves with the increase in decorrelation distance (i.e., less intermittent field). The OK interpolation technique uses more prior/auxiliary information and correlates slightly better with ground truth than IDW, and the performance of OK and IDW-based algorithms with multiple points representing a CML is slightly better than that with only one point representing a CML. D’ Amico et al. [47] applied the tomography technology to reconstruct the two-dimensional rainfall accumulation field, and it was found that the accuracy of the reconstruction algorithm can be improved by increasing the network density. For the reconstruction of 2-D rainfall field, Gazit et al. [72] described the statistical characteristics of the measurements and then used the compressed sensing (CS) theory, i.e., phase transition diagram, to solve the rain-field sparsity problem.

### 4.6. Uncertainties Analysis

Every step of CML-based rainfall retrieval may introduce errors. In order to further improve the accuracy of rainfall measurement, it is necessary to analyze the sources of errors and their impacts.

#### 4.6.1. Uncertainties in Each Step of Signal Processing

- In wet/dry classification, whether adopting multiple CMLs or single CML RSL data, the empirical-based thresholds for classification will certainly lead to uncertainties and errors.
- In baseline determination, the baseline in rain period determined by the NLA method is constant. Yet, some signal fluctuations, such as the fluctuations during the dry period, may also occur during the rain period. For shorter links or lower frequencies links, the natural fluctuation of baseline attenuation has the same order of magnitude as the quantization interval (1 dB) [1].
- In WAA compensation, the WAA value depends on the CML antenna characteristics (hydrophobicity or hydrophilicity) and the weather environment. For example, the water vapor condensation induced by the temperature drop at night, even though there is no rain, can cause the WAA value to be higher than that in light rain. In general, the WAA value increases as the rainfall intensity becomes stronger. The WAA effect on CML-based rainfall retrieval is probably the major source of errors for short links, because WAA becomes more comparable to the overall link attenuation as the length of the link decreases.
- In rain rate calculation, the *k-R* power law relationship in Equation (1) is approximately linear in the frequency of 20–35 GHz, but when the frequency is lower or higher than that, the uncertainty caused by DSD increases [73]. Rain rates calculated by different sampling strategies and time resolution also have deviations [74]. Generally, the performance of rainfall measurement using minimum/maximum RSL with time resolution of 15 min is better than that using the instantaneous RSL. On the other hand, for different time resolutions, due to the spatial and temporal variability of rainfall, a longer sampling time interval (e.g., 15 min) will lead to a larger error, but a very short sampling time interval (e.g., 1 s) can increase the accuracy while also increasing the computational complexity.
- In rainfall map generation based on the interpolation algorithm, errors and uncertainties in the reconstructed rainfall field increase as the time aggregation decreases and the distance between two CMLs increases. The uncertainties in daily rainfall map are lower than the 15 min rainfall map, because the errors in the 15 min rainfall map are aggregated to cancel each other out over the course of a day [75]. The OK interpolation algorithm utilizes the average path link rainfall data, i.e., the mid-point rainfall data in the link, and so converting the line scale to the point scale will produce errors, which is called interpolation uncertainty [53].

#### 4.6.2. Other Sources of Errors

The characteristics of microwave links and monitoring environment will also induce errors and affect the overall measurement accuracy. De Vos et al. [76] used seven months of instantaneous signal power collected from about 2000 microwave links in the Netherlands to retrieve rainfall intensity, and the results show that the bias is the relatively high for path lengths less than 2 km during late night, early morning, and colder months. Van Leth et al. [77] installed three microwave links between two main buildings in Wageningen, with one commercial microwave link at 38 GHz and two research microwave links at 26 GHz and 38 GHz, respectively. The results show that WAA in the presence of fog or dew induces about 3 dB attenuation, and changes in temperature can also cause an attenuation of similar magnitude.

Rios Gaona et al. [75] divided the errors presented in rainfall maps, which were generated from link rainfall depths, into two categories: (1) measurement errors, including sampling interval, received power quantification, wet/dry classification, baseline fluctuation, WAA, DSD, multipath, etc., and (2) interpolation errors, including interpolation algorithm and link space density. An extensive experiment was carried out by comparing rainfall maps created from three sets of link rainfall depths: actual available links, simulated links with the actual network availability, and simulated links with 100% network availability assumed. The results show that the uncertainty is mainly caused by measurement errors. The factors leading to the error of CML-based rainfall measurement are ranked from most important to least important by Messer et al. [1], which are spatial variability of rainfall, zero baseline selection, DSD and WAA, and RSL quantification.

## 5. Machine Learning for CML-Based Rainfall Measurement

Through the uncertainty analysis, we can find that it is a very complicated process from RSL raw data acquisition to the generation of rainfall maps, and many factors and uncertainties may deteriorate the accuracy of rainfall measurement. In recent years, many works have applied machine learning algorithms to evaluate the relationship between the microwave link attenuation and rain rate, thus avoiding the tedious analysis of uncertainty sources and error quantization.

### 5.1. Application of Machine Learning

Machine learning focuses on finding patterns in data and uses these patterns to make predictions [78,79,80]. Table 3 lists the machine learning algorithms applied in microwave-link-based rainfall measurement during recent years. For wet/dry classification, He et al. [81] analyzed the variation of RSL data of C-band microwave links under the conditions of no rain, drizzle, light rain, and moderate rain; then the LSTM network was used to analyze the RSL data in different time scales to distinguish the rainfall period. Polz et al. [82] established a CNN-based model by using the instantaneous RSL data collected from 3904 CMLs in Germany for wet/dry classification. Four months of data from 800 randomly selected CMLs are used to train, and two different months of data, one for all CMLs and one for the 3104 CMLs not included in the training, are used for model verification. Radar data adjusted by RGs are used as the reference method. The results show that the designed CNN model is superior to the current state-of-the-art model, which uses the rolling standard deviation of the CML signal as the detection criterion.

For WAA quantization, Pu et al. [83] obtained the WAA values based on LSTM, which has a good correlation with the measured WAA, and the performance of the model in the 72.75 GHz link is better than that in the 82.75 GHz link. For accurate rain rate calculation, Pudashine et al. [84] designed and trained a DL model by using RGs data and applied it to CML-based rainfall measurement, and the result is better than the constant weighted average method. Liu et al. [85] used the measurement report (MR) data in a time-division–long-term-evolution (TD-LTE) network to retrieve rainfall, and support vector classification (SVC) and artificial neural network (ANN) were used to distinguish wet/dry weather and estimate rain rate, respectively. Diba et al. [86] compared the accuracy of rainfall measurement between the terrestrial links (18, 38, 75 GHz) and the satellite links (12.25 and 20.74 GHz). The accuracy of rainfall measurement by applying ANN and LSTM at 11 GHz terrestrial link is studied, and the results show that LSTM is better than ANN.

In addition, machine learning can also be used to distinguish different types of rainfall (convective or stratiform) [87], determine the baseline [88], predict the short-term attenuation [50], and so on.

### 5.2. Potential of Deep Learning

It can be found that DL algorithms, such as CNN, RNN, LSTM, and GRU, are widely used in CML-based rainfall measurement compared with the traditional machine learning algorithms. This is because traditional machine learning has limited ability to simulate high spatio-temporal dynamics events such as rainfall, while DL can capture high-dimensional and multi-modal data distribution, automatically extract spatio-temporal features, and better mine the internal logic of data [89].

#### 5.2.1. LSTM

**Figure 4:**
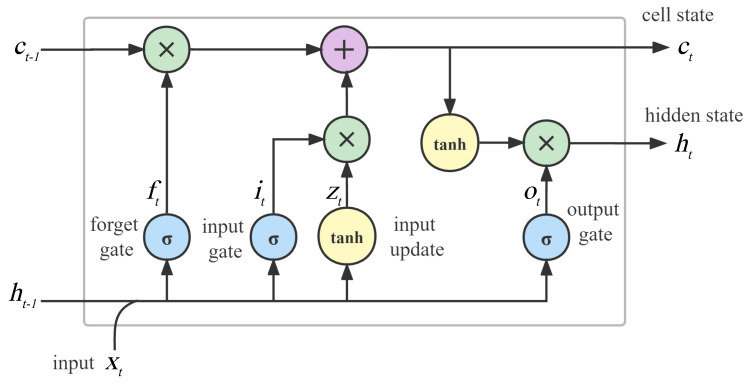
LSTM cell structure.

As one of the most popular DL algorithms for processing time-series data, the LSTM is equipped with a memory cell and several gates to overcome the gradient explosion or gradient disappearance problem of RNN. Figure 4 shows the cell structure of the LSTM, and Equations (10)–(15) give the basic mathematical computational steps of the algorithm [90]. First, the forgetting gate $f_{t}$, input gate $i_{t}$, and candidate value $z_{t}$ are calculated by using the current input $x_{t}$ and the previous hidden state $h_{t-1}$. Second, the current memory cell state $c_{t}$ is updated by combining $f_{t}$, $i_{t}$, $z_{t}$, and the previous memory cell state $c_{t-1}$. Finally, the information of $c_{t}$ is transferred to the current hidden state $h_{t}$ through the output gate $o_{t}$.

(10) $$f_{t}=\sigma \left(W_{f}x_{t}+U_{f}h_{t-1}+b_{f}\right),$$

(11) $$i_{t}=\sigma \left(W_{i}x_{t}+U_{i}h_{t-1}+b_{i}\right),$$

(12) $$z_{t}=tanh(W_{z}x_{t}+U_{z}h_{t-1}+b_{z}),$$

(13) $$c_{t}=i_{t}\times z_{t}+f_{t}\times c_{t-1},$$

(14) $$o_{t}=\sigma \left(W_{0}x_{t}+U_{0}h_{t-1}+b_{0}\right),$$

(15) $$h_{t}=o_{t}\times tanh(c_{t}),$$

where σ(·) and tanh (·) are the sigmoid and hyperbolic tangent functions, respectively. The weight matrices $W_{f}$, $W_{i}$, $W_{z}$, $W_{o}$, $U_{f}$, $U_{i}$, $U_{z}$, $U_{o}$ and the bias vectors $b_{f}$, $b_{i}$, $b_{z}$, $b_{o}$ can be predicted in the training stage. Through the cooperation between the memory cell and the gates, LSTM is equipped with a powerful ability to predict time series with long-term dependence.

#### 5.2.2. GRU

**Figure 5:**
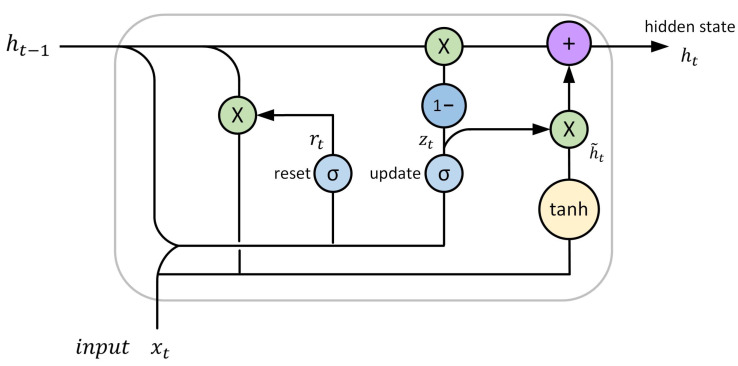
GRU structure.

GRU is proposed in [91] and its structure is simpler as compared to LSTM. It eliminates memory cell state and integrates the forgetting and input gates into one update gate to simplify the structural model. Figure 5 shows the structure of the GRU, and Equations (16)–(19) give the basic mathematical computational steps of the algorithm [92]. First, two gate states named reset gate $r_{t}$ and update gate $z_{t}$ are obtained from the previous state $h_{t-1}$ and the current input $x_{t}$. Second, $h_{t-1}$ × $r_{t}$ is spliced with input $x_{t}$, and then through a tanh activation function to covert the data into the range of −1∼1; that is, $\tilde{h_{t}}$ is obtained. Finally, the current hidden state $h_{t}$ is obtained through the update gate $z_{t}$.

(16) $$z_{t}=\sigma \left(W_{z}\cdot \left[h_{t-1},x_{t}\right]\right),$$

(17) $$r_{t}=\sigma \left(W_{r}\cdot \left[h_{t-1},x_{t}\right]\right),$$

(18) $$\tilde{h_{t}}=tanh(W\cdot \left[r_{t}\times h_{t-1},x_{t}\right]),$$

(19) $$h_{t}=\left(1-z_{t}\right)\times h_{t-1}+z_{t}\times \tilde{h_{t}}.$$

where $W_{z}$, $W_{r}$, and *W* are weight matrices. In the GRU model, the reset gate defines how to combine new input with previous memories, and how much memory should be retained is decided by the update gate. Some varaints of GRU have been proposed and evaluated by reducing parameters in the update and reset gates [93]. Considering the hardware computing power and time cost, the more practical GRU is suggested to be chosen. However, if training data are sufficient, LSTM may generate better predictions due to its strong expressive ability. So, which model to use depends on the system requirement. The GRU model has been used in CML-based rainfall measurement (e.g., [65]), which we have discussed in Section 4.4.

## 6. Monitoring Phenomena Other than Rain

The attenuation of microwave links can not only be used to measure rainfall, but also theoretically monitor all substances that affect microwave signals propagating in the atmospheric environment, such as water vapor, oxygen, CO_2_, snow, dust, and so on [94,95]. However, different substances have different absorption characteristics for specific frequency bands. For example, water vapor strongly absorbs the microwave in the frequency around 22.2 GHz (i.e., 1.35 cm in wavelength), and oxygen has a strong absorption of microwave in the frequency around 60 GHz (i.e., 0.5 cm in wavelength). Therefore, when measuring different atmospheric substances, the corresponding absorption bands should be avoided. Monitoring other meteorological phenomena by microwave link attenuation is summarized as follows:

### 6.1. Water Vapor

Zheng et al. [96] used a 4.8 km long E-band mm-wave link in the Xianghe area located in the city of Langfang, Hebei Province, China. As compared to the data of meteorological station, the annual correlation value of the water vapor retrieved from the link is as high as 0.95, RMSE is as low as 0.35, and the average relative error is as low as 0.05. Pu et al. [97] proposed a water vapor retrieval model by using dual-frequency E-band CMLs based on LSTM network, and the results show that the retrieved water vapor density is in good agreement with the results measured by temperature and humidity sensors. Fencl et al. [98] also used a longer E-band link to realize the water vapor retrieval because E-band is more sensitive to raindrops and atmospheric gases, and the signal attenuation is sufficiently strong to enable the detection of water vapor at long CMLs. The proposed empirical model does not require in situ calibration. However, the separation of gaseous attenuation from the total losses is more challenging than traditional 15–40 GHz CMLs.

### 6.2. DSD

Van Leth et al. [99] used two or three configured microwave link instruments to estimate the three parameters of a gamma DSD model; the DSD retrieval performance using different microwave link combinations of frequency and polarization are analyzed. The experimental results show that the DSD retrieval based on microwave link is accurate under ideal conditions, and the accuracy and successful rate in practical situations are highly dependent on the stability of the power level and the precision of the instrument. Song et al. [100] proposed a method for retrieving path-averaged DSD parameters using dual-frequency and dual-polarization joint microwave links. The DSD parameters are obtained based on the Levenberg–Marquardt optimization algorithm, and this method can be used as an effective supplement to traditional DSD monitoring systems, such as disdrometer.

### 6.3. Hydrometeor Types

Pu et al. [101] proposed a method to identify rain, graupel, and wet snow based on microwave links, and single-frequency, dual-frequency, and triple-frequency models are established by using the extreme learning machine algorithm to analyze the hydrometeor size distribution data in Nanjing. The results indicate that accuracy increases as the total frequency or frequency difference among microwave links increases.

When using CML to measure rainfall in stormy weather, the wind will cause the antenna to shift slightly, resulting in the RSL fluctuations. Atmospheric dust particles and vehicle emissions (e.g., CO and NO_x_) can also induce microwave signal attenuation and phase shift. Hence, it seems feasible to monitor wind, dust and air pollution by measuring the attenuation or phase change caused by these small changes.

## 7. Hydrological Application

Rainfall measured by CML has a broad application prospect in radar rain attenuation correction, urban rainfall-runoff simulation, drainage pipe flow prediction, flash flood warning, and so on [102,103]. On the one hand, CML-based measurement provides a new source of rainfall data for hydrology research; on the other hand, the results of hydrological application can verify the performance of CML-based rainfall measurement.

### 7.1. Combined with Conventional Methods for Rainfall Measurement

Zhang et al. [104] verified that microwave links in any direction can be used to compensate the attenuation effect of radar reflectivity through experimental methods. A new method of CML-based rainfall retrieval adjusted by RGs is proposed by Fencl et al. [105], which does not require installing RGs near the CMLs intentionally. Using RGs with different spatial and temporal resolutions, even if they are relatively far away, can improve the performance of CML-based rainfall measurement. Bianchi et al. [44] integrated the RG, radar and microwave links to improve the measurement accuracy of the spatial distribution of rainfall and rainfall intensity, and the Gauss–Newton method is used to minimize the cost function of all sensing methods. CML also has a great potential in the calibration of rainfall measured by radar or satellite images [106].

### 7.2. Runoff Simulation and Prediction

Rainfall is the key input of many hydrological models, and the errors and uncertainties of rainfall data sets will propagate through the hydrological system [107,108]. Smiatek et al. [109] used CML-derived rainfall data as inputs of a distributed hydrological water balance model, named as WaSiM-ETH, to predict the runoff in the Ammer River Basin in Germany. As compared to RG and radar, the Nash–Sutcliffe efficiency (NSE) based on the CML data model is much higher, which effectively improved the initial state of the flow simulation and forecasting system. Liu et al. [85] used a lumped hydrological model, i.e., MISDc, to simulate the runoff of Andunshui River basin in Huizhou, Guangdong Province, China, with the CML rainfall data as the inputs, and the model also generates a high NSE.

### 7.3. Urban Drainage System Scheduling

It is important to predict the ability of an urban catchment to respond to rainfall-generated runoff, which is essential for effective management and control of urban drainage systems. Fencl et al. [45] verified that the application of CML-based rainfall measurement data in the urban hydrological model can better capture the spatial and temporal dynamic distribution of rainfall, and thus better capture the temporal dynamic changes of drainage pipe flow, especially in heavy rainfall, which can better reflect the changes of outlet and peak flows. Pastorek et al. [110,111] studied the effects of the characteristics and locations of CMLs on the runoff forecast. The experimental results show that the quantitative precipitation estimates (QPEs) of shorter CMLs located within or near the catchment boundary reproduce the runoff dynamics better than the QPEs of longer CMLs located outside the catchment boundary.

### 7.4. Flash Flood Warning

Accurate and real-time rainfall measurement plays an important role in flash flood warning [112,113]. A flash flood early warning system using rainfall intensity data, which are retrieved from terrestrial CMLs and the geostationary satellite in Kenya, Africa, is designed by [114], and it has been proven to be an effective measure to strengthen the resilience to climate change of developing countries. CMLs are sparsely deployed in mountainous areas and the link length is relatively long; rainfall measured by CML is the line average of the whole path, and high local rainfall intensities are smoothed, i.e., the peak rainfall intensities can easily be ignored. To resolve this problem, Eshel et al. [115] proposed a new method to identify the potential conditions of flash floods using only a single long microwave link integrated with weather radar. Radar measurement is first used to identify rainfall changes along the link path, and then a CML is used to quantify the rain rate. The results show that there is a close relationship between the proposed inverse-kurtosis–rain-rate relation and the flash flood response, and thus flash flood warning systems can possibly be improved.

## 8. Challenges and Future Directions

### 8.1. Development of CML Data Acquisition Standards

The feasibility of using CML to measure rainfall has been verified by works and projects, but the difficulty of obtaining available CML data in WCNs is obvious, thus limiting the adoption of this technology [19,116]. Although some studies have published open-access datasets, as listed in Table 4, due to security considerations, the way to obtain CML sensing data is still greatly limited, especially the CML datasets from the Asia-Pacific region, most of which are not public available to the best of our knowledge. The original intention of obtaining CML data is to improve wireless communications business, and using CML data for sensing requires complicated signal analysis and processing, i.e., we need to transform the rain-induced attenuation data, which are noise for communication, into useful information for sensing. It is suggested that relevant standard protocols for microwave-based fronthaul/backhaul communications should be standardized to facilitate not only communications but also sensing and attenuation data acquisition. By achieving this, the barriers between scientific research and commercial business can be overcome, and the CML-based rainfall measurement can be widely adopted to benefit the society as a whole.

### 8.2. Strengthen Uncertainty Analysis

Uncertainty analysis is an important prerequisite for formulating effective measures to improve the measurement accuracy. The spatio-temporal dynamics of rainfall, multi-path propagation, atmospheric absorption, sampling strategy, and quantization error are all sources of uncertainty. The combination of data resources, computing power, and machine learning provides an opportunity to discover the spatial and temporal uncertainties of the rainfall and the relationship between the rain-induced attenuation and rainfall intensity. Moreover, the input–output relationship derived from these data-driven models can provide inspiration and guidance to explain and understand the mechanism in the physical world. In addition, for uncertainty study, we can also build a dedicated microwave link experimental environment to carry out targeted analysis for a certain interference factor. Because the CML network covers a wide area, and the amount of CML data is huge, many of the uncertainties are difficult to quantify when the CML data are directly applied to actual rainfall measurement.

### 8.3. Extending Machine Learning Capabilities

Machine learning has a great application potential in the field of earth environment systems [121]. In particular, DL can extract the spatio-temporal structure and characteristics of data, and it is a good solution to the problem of strong time dependence such as rainfall simulation and prediction. However, machine learning also has some challenges, such as the cost of big data and interpretability [122,123]. The cost of a big data platform is high, and only when the cost of data collation and cleaning is low can the advantages of big data be maximized. Machine learning lacks behavioral interpretation. Therefore, in the future, we should try to give machine learning the necessary causal reasoning ability to get rid of the traditional “black box” operation [124].

### 8.4. Assimilation of Multi-Source and Heterogeneous Data

Data assimilation is always an important problem in the data-intensive field. The data assimilation problem in the study of CML-based rainfall measurement is mainly reflected in two aspects: one is the data assimilation among different CMLs. One CML can measure the average rain rate along the path, and if we want to get the surface rainfall information in a larger coverage area, we need multiple CMLs nearby to cooperate with each other. However, different CMLs use different carrier frequencies, sampling strategies, and power resolutions, so it is necessary to convert the data of various formats collected by multiple independent CMLs into data of the same magnitude and uniform specification. On the other hand, data collected from different rainfall monitoring methods, i.e., RGs, radar, satellite, and CMLs, need to be assimilated. CML-based rainfall measurement can be used as a supplement to the existing rainfall measurement methods [125], and for a specific monitoring area, using multiple rainfall measurement methods may be able to construct a more detailed rainfall map than using only one of them.

### 8.5. WAA Quantization

WAA has been experimentally verified to have an important influence on the rainfall intensity retrieval, and many factors affect the WAA value, such as rainfall intensity, temperature, link length, radome material, and so on. The WAA value should be a dynamic function of time, and it is obviously inappropriate to give WAA a fixed constant to compensate the total attenuation. In the future, we should further understand the change pattern of WAA and then use appropriate time-frequency domain transformation, probability statistics, machine learning, and other methods to construct WAA model.

### 8.6. Refinement of Rainfall Retrieval and Mapping Algorithms

When the rainfall intensity is strong, the attenuation of microwave signal is obvious, and the classification of wet/dry weather, the determination of zero baseline, and the calculation of rain rate are more accurate. On the contrary, when the rainfall intensity is light, the attenuation of microwave signal caused by rain is easily covered by abnormal signal fluctuations or WAA, and uncertainty is greater compared with heavy rain, so the calculation method of rain rate should be refined according to different rainfall intensities. It can be combined with other measurement methods to achieve more accurate rainfall measurement. The network density and topology of microwave links have a great influence on the construction of rainfall map [126]. In general, the higher the density, the higher the resolution of the constructed rainfall field. The design of CML topology should refer to the topology optimization algorithms of wireless sensor networks (WSNs) to further expand the sensing range and improve the sensing accuracy. Based on the existing GIS spatial interpolation and tomographic reconstruction techniques, more flexible algorithms of rainfall mapping should be designed to meet the complex geographical conditions.

### 8.7. Adoption of Synthetic Storm Technique

Synthetic Storm Technique (SST) is one of the most reliable methods to estimate rain attenuation time series [127]. Rain attenuation time series are usually generated through SST with the input rain rate data measured by RGs or other equipments. Based on the generated rain attenuation time series, the seasonal, annual, or daily statistics which reflect the dynamic patterns of the received power of the signal in a communications link are obtained, and then the rain attenuation can be compensated more effectively. The implementation of the SST technique can yield more accurate predicted values of rain attenuation as compared to the ITU model [128,129]. On the other hand, SST may also be used to estimate the rain rate in short time intervals (e.g., 1 min) by inverting the convolution integral. Therefore, SST should be considered and given more attention in the future in the CML-based rainfall measurement field.

### 8.8. Exploring Information Other Than RSL as the Basis of Retrieval

At present, most of the studies on CML-based rainfall measurement use the maximum–minimum RSL, average RSL, instantaneous RSL, and other amplitude informations, and a few studies have used other informations, for example, the transmission error of a microwave link signal was used for the first time by Habi et al. [130] to divide dry and wet weather. Pu et al. [87] proposed a rainfall type distinguishing method by using the differential attenuation rate from multi-frequency and dual-polarization microwave links. In the research of using WIFI signals to identify indoor human activities, the channel state information (CSI) with phase information is commonly used as the retrieval basis. Therefore, CML-based rainfall measurement should also be considered to explore some phase information other than RSL as the basis of retrieval, which may be able to obtain more accurate and real-time rainfall spatial information.

### 8.9. Promoting the Integration of Sensing and Communications

The proposal of sensing and communication integration provides a new development opportunity for CML-based rainfall measurement. On the one hand, monitoring rainfall by using CMLs is a typical application of communication-assisted sensing, especially driven by the deep integration of information and communication technology (ICT), AI, and BD technologies. The absorption spectrum characteristics of water molecules based on 6G terahertz and the “fingerprint spectrum” characteristics of chemical information will achieve more accurate and wide-area real-time monitoring of rainfall, atmospheric humidity, and air quality in the near future. On the other hand, CML-based rainfall sensing can also assist the promotion and development of communication services. For example, sensing technologies such as beamforming can be used to assist the transmitting terminal of each node in the communication system to realize the parameter set selection or parameter configuration for transmission signals according to real-time environmental conditions, and thus to save energy, improve spectrum utilization, and so on. Therefore, in the future, the abilities of sensing and communications should be optimized jointly to realize the overall performance improvement.

## 9. Conclusions

As a new approach of rainfall measurement, The CMLs in wireless cellular networks have a broad application prospect. In densely populated cities, CMLs can be used as an effective supplement to traditional rainfall measurement methods. Moreover, in remote mountainous areas, where RGs and radars are not easy to deploy and maintain, CMLs have much greater potential in some scenarios, such as real-time rainfall monitoring, short-term rainfall forecasting, flash flood warning, and so on. During the more than ten years of development of CML-based rainfall measurement technology, great progresses have been made in the accuracy of wet/dry classification, the real-time dynamics of baseline determination, the authenticity of WAA simulation, the diversity of rainfall intensity retrieval and mapping algorithms, and so on. More and more researchers around the world have devoted themselves to this research.

Although CML-based rainfall measurement has been greatly improved in terms of both monitoring range and mapping accuracy, there are still some challenges that limit the practical application of this technology. The biggest problem is the data acquisition. Because the operation processes of cellular networks for communications and sensing are independent from each other, it is relatively difficult to obtain the dedicated communication data and adapt them to suitable environmental sensing data. From the view of technological aspects, various error sources have a certain impact on the accuracy of rainfall measurement, especially the change in WAA. Therefore, dedicated microwave link experimental equipment should be built to further analyze the weights of various errors in the total errors, and thus to provide help for acquiring more accurate rainfall information in actual CML network environments.

In recent years, machine learning has been widely used in CML-based rainfall measurement to solve problems mostly related to big data, such as classification, regression, simulation, prediction, and so on. In particular, deep learning provides a new way to simulate the complicated hydrological cycle processes. In the future, we should strengthen the combination of data-driven machine learning and physical mechanism models in order to explain and understand the data logic. It is also necessary to enhance the cooperation among different rainfall measurement methods to establish a more comprehensive rainfall measurement network. At the same time, applying CML-based rainfall measurement data to hydrological cycles should be promoted, and the potential of CMLs as an opportunistic wireless sensor network in environmental sensing should be fully explored. This paper has summarized the state-of-the-art of CML-based rainfall measurement technology and discussed the future directions, and we hope it can inspire more innovative ideas and deeper thinking.

## Figures and Tables

**Table 1:** Comparisons of conventional and CML-based rainfall measurement techniques.

Techniques	Advantages	Disadvantages
Rain Gauge	High accuracy	Point measurement; low spatial resolution; high capital and operational cost; difficult to deploy in mountainous areas
Weather Radar	Broad spatial coverage of up to 300 km	Low accuracy in near-surface measurement; easy to be affected by ground obstacles at a low elevation angle
Satellite	Global scale	Coarse resolution for small spatial and temporal scales; affected by clouds; high time lag
Commercial Microwave Links	Path-integrated and near-surface measurements; high spatial and temporal resolution; no additional capital cost	Hard to acquire CML data; relatively high complexity for data processing

**Table 2:** Countries that have used CML to measure rainfall.

CML Data							
Authors (Year)	Country	Frequency (GHz)	Link Number	Length (km)	Temporal	Quantization Level (dB)	Remarks
Messer et al. (2006) [39]	Israel	—	—	—	15 min	—	The correlation between rainfall intensity measured by CML and RG is 0.86 for a 15 min interval and 0.9 for an hourly interval.
Leijnse et al. (2007) [41]	The Netherlands	38	2	7.75, 6.72	15 min	1	Eight rainfall events are evaluated, and the results are consistent with the rainfall retrieved from RGs and C-band radar.
Schleiss et al. (2010) [42]	France	26, 19	4	3.7, 3.7, 7.1, 2.4	30s, 6s	1	A wet and dry weather classification method is proposed, which can identify 92% of all rainy periods and 93% of the total rain amount.
Chwala et al. (2012) [43]	Germany	15, 18.7, 23	5	17.4, 10.2, 4, 17.1, 10.4	selectable	<0.05	A new algorithm based on short-time Fourier transform (STFT) is proposed for the wet/dry classification. The correlation reaches 0.81 for the link-gauge comparison.
Bianchi et al. (2013) [44]	Switzerland	23, 38, 58	14	0.3–8.4	5 min	0.1 or 1	RGs, weather radar and CMLs are combined to estimate the intensity and temporal distribution of rainfall more accurately.
Fencl et al. (2013) [45]	Czech Republic	38	14	—	—	—	CML networks can better capture the spatio-temporal rainfall dynamics, especially in heavy rain, and thus improve pipe flow prediction.
Doumounia et al. (2014) [46]	Burkina Faso	7	1	29	1s	1	95% of the rainy days are detected by CML measurement, and the correlation with the RGs data series is 0.8.
D’Amico et al. (2016) [47]	Italy	25	3	average of 6	—	—	Tomographic technique was applied to reconstruct 2-D fields of rainfall accumulation, and the link density and topology affect the accuracy of the reconstruction algorithm.
Rios Gaona et al. (2018) [48]	Brazil	above 15	145	shorter than 20	—	0.1	As compared to RGs, CML-based measurement can better capture the city-average rainfall dynamics.
Sohail Afzal et al. (2018) [49]	Pakistan	38	35	0.5–2.5	15 min	—	The correlation coefficient value between rainfall intensity measured by CMLs and RGs is as high as 0.97.
Jacoby et al. (2020) [50]	Sweden	14–39	17	1.5–7	10 s	—	Using long short-term memory (LSTM) to learn from previous attenuation values is sufficient to generate accurate attenuation predictions.
Song et al. (2021) [51]	China	15–23	8	0.55–1.08	1 min	0.1	The correlation coefficient values between the rain rate measured by CMLs and RGs are all higher than 0.77, and the highest coefficient is over 0.9.
Pudashin et al. (2021) [52]	Australia	10–40	144	0.2–57	15 min	0.1	Using two types of datasets collected by different sampling strategies (maximum/minimum RSL and average RSL) to retrieve rainfall, the results show that the maximum/minimum RSL data are better than average in terms of the statistics, i.e., root mean square error (RMSE), bias, and coefficient of variation (CV).

"—" represents that the content is not mentioned in the literature.

**Table 3:** Machine learning algorithms applied in CML-based rainfall measurements.

Ref.	Algorithms	Function	Data Source	Data for Training and Testing	Results
[81]	LSTM	Wet/dry classification	Experimental data were collected from 1/11–31/12 except for 13/12–21/12 by using a C-band microwave link (7.7 GHz)	Data from 1/11–30/11 are used to train a classifier, and the December data are used for testing.	The accuracy of wet/dry classification is higher than 60%, and even higher than 98% in some days.
[57]	SVM	Wet/dry classification	15 microwave links (15–23 GHz) and 8 RGs	Half of the data from rainfall time over 2 h in 14 days were used as the training set and the remaining half as the test set.	The accuracy of rainfall identification is higher than 80%, and most of the accuracy is even higher than 90%.
[82]	CNN	Wet/dry classification	Data came from 3904 CMLs, and gauge-adjusted radar data are used as a reference	4 months of data from 800 randomly selected CMLs were used for training and 2 different months of data for testing.	76% of rainfall and 97% of non-rainfall periods can be detected, and more than 90% of rainfall intensities that are greater than 0.6 mmh^−1^ can be detected.
[83]	LSTM	WAA quantization	Total attenuation data of 6 E-band full-duplex CMLs and 4 RGs data	Rain period data were divided into 12 subsets, of which 10 subsets were training sets and the remaining two for testing.	It has a good correlation with the RGs measured WAA, but the cumulative rainfall estimates based on LSTM are lower when the rainfall increases sharply.
[84]	LSTM	Rain rate estimation	A CML (22.715 GHz) and an OTT PARSIVEL disdrometer	The training group accounts for 80% of the whole sequence, and the remaining 20% is used as testing group.	The relative bias decreases from 7.39% to 1.14%, and the coefficient of determination (R^2^) increases from 0.71 to 0.82 compared with constant weighted average method.
[65]	GRU-RNN	Rain rate estimation	A total of 1.4M samples are from 40 full duplex links and 8 RGs in Swedish region, and 1.7M samples are from 34 full duplex links and 9 RGs in the Israeli region	80% of the total samples are used as training set and the remaining 20% as validation.	RMSE and bias are smaller compared with the traditional power-law-based algorithm, and the trade-off between performance and robustness of RNN methods can be controlled by introducing a TN layer.
[85]	SVC, ANN	Wet/dry classification, rain rate estimation	Measurement report (MR) data from TD-LTE networks, and RGs data and runoff data are used as references	60% of the wet/dry records are used as ANN training samples for classification, while the remaining 40% are used as testing samples.	The performance of rainfall retrieval from MR data is in good agreement with RG measurements, and the accuracy is more than 80% in the application of runoff simulation.
[86]	ANN, LSTM	Rain rate estimation	3×216480 RSL units and 2164800 target rain rate samples in Korea region, and satellite RSL data in Ethiopia region	Data are split into 85% and 15% for training and testing.	Rainfall retrieval performance of ground link is better than that of satellite link. Performance (RMSE, R^2^, CC) of LSTM at 11 GHz ground link is better than that of ANN.
[87]	DT, PNN, GDA, LR	Rainfall types classification	2475 samples of convective rainfall (31.3%) and 5441 samples of stratiform rainfall (68.7%) from March to November	7916 total samples are divided into 5 groups on average, 4 groups are selected as the training set, and the remaining 1 group is used as the test set.	DT and PNN algorithms have better fault tolerant ability than GDA and LR, and the classification accuracies of tri-frequency models are higher than those of dual-frequency models.
[50]	LSTM	CML attenuation prediction	17 CMs with the frequencies of 14–39 GHz	1400 h of training time; 16 h of validation time.	The prediction accuracy of CML attenuation values by LSTM during rainfall is greater than ARIMA.

**Table 4:** Open-access datasets.

Dataset	Code Availability	Location	Data Description	URL
Dübendorf data [117]	No	Dubendorf, Switzerland	Received and transmitted power of 1 dual-polarization CML (38 GHz); rainfall rate and cumulative rainfall from 5 RGs; temperature, dew point, relative humidity, wind direction, and wind speed from 5 weather stations.	https://doi.org/10.5281/zenodo.4923125 (accessed on 17 May 2022)
Wageningen data [77]	No	Wageningen, the Netherlands	Received power of 1 CML (38 GHz) and 2 research microwave links (26 GHz, 38 GHz); relative humidity, temperature, and wind speed from 5 disdrometers.	https://doi.org/10.4121/uuid:1dd45123-c732-4390-9fe4-6e09b578d4ff (accessed on 17 May 2022)
Melbourne data [3]	No	Melbourne, Australia	RSL data from a microwave research link (24 GHz), and specific attenuation, wind speed and direction, air temperature and humidity, barometric pressure, and so on from disdrometers, RGs, and weather station.	https://doi.org/10.5281/zenodo.4442322 (accessed on 17 May 2022)
PSO data [118]	No	The Netherlands	Frequency, minimum and maximum received power, path length, coordinates, and link ID of about 2800 microwave sublinks; rain intensity from gauge-adjusted radar.	https://doi.org/10.4121/uuid:323587ea-82b7-4cff-b123-c660424345e5, https://dataplatform.knmi.nl/catalog/datasets/index.html?x-dataset=rad_nl25_rac_mfbs_5min&x-dataset-version=2.0 (accessed on 17 May 2022)
Sri Lanka data [119]	No	Sri Lanka	The gridded rainfall maps retrieved from CML data from Sri Lanka over the 3.5 month period, and hourly/daily rainfall depths from satellite product and the global precipitation measurement (GPM) product.	https://doi.org/10.4121/14166539.v2,https://gpm.nasa.gov/data/directory (accessed on 17 May 2022)
R package “RAINLINK” [53]	Yes	The Netherlands	Frequency, maximum RSL, minimum RSL, link length, location coordinates of about 2600 CMLs.	https://github.com/overeem11/RAINLINK (accessed on 17 May 2022)
			Code processing steps: data preprocessing, wet/dry classification, baseline determination, filtering of outliers, correction of received power, path-average rainfall intensity estimation, generation of rainfall map, and map visualization.	
Prague data and code [34]	Yes	Prague, Czech Republic	Total power loss of 6 E-band full-duplex CMLs; rainfall intensity, temperature, and humidity from 4 RGs.	https://doi.org/10.5281/zenodo.4090953 (accessed on 17 May 2022)
			Code processing steps: data preprocessing, loading data, RG-based wet/dry classification, estimating baseline, quantifying WAA, estimating rainfall, quantifying uncertainty, and retrieving water vapor density.	
Python package “pycomlink” [120]	Yes	Germany	Code processing steps: data sanity checks, anomaly detection, wet/dry classification, baseline calculation, wet antenna correction, transformation from attenuation to rain rate, rainfall map generation, and results validation against RGs.	https://github.com/pycomlink/pycomlink (accessed on 17 May 2022)

## Data Availability

Not applicable.

## Abbreviations

The following abbreviations are used in this manuscript:

AI	Artificial intelligence
ANN	Artificial neural network
ARIMA	Auto-regressive integrated moving average
BD	Big data
CML	Commercial microwave link
CNN	Convolutional neural networks
CS	Compressed sensing
CSI	Channel state information
CV	Coefficient of variation
DL	Deep learning
DSD	Drop size distribution
DT	Decision tree
GDA	Gaussian discriminant analysis
GIS	Geography information system
ICT	Information and communication technology
IDW	Inverse distance weighting
ITU-R	Radiocommunication sector of International Telecommunication Union
LR	Logistic regression
LSTM	Long short-term memory
MR	Measurement report
NLA	Nearby link approach
NSE	Nash–Sutcliffe efficiency
OK	Ordinary kriging
PNN	Probabilistic neural network
QoS	Quality of service
QPE	Quantitative precipitation estimation
RG	Rain gauge
RMSE	Root mean square error
RNN	Recurrent neural network
RSL	Received signal level
SST	Synthetic storm technique
STFT	Short-time Fourier transform
SVM	Support vector machine
TD-LTE	Time division long-term evolution
TN	Time normalization
TRMM	Tropical rainfall measuring mission
WAA	Wet antenna attenuation
WCN	Wireless cellular network
